# Single-cell RNA sequencing analysis of human bone-marrow-derived mesenchymal stem cells and functional subpopulation identification

**DOI:** 10.1038/s12276-022-00749-5

**Published:** 2022-04-01

**Authors:** Zhongyu Xie, Wenhui Yu, Guiwen Ye, Jinteng Li, Guan Zheng, Wenjie Liu, Jiajie Lin, Zepeng Su, Yunshu Che, Feng Ye, Zhaoqiang Zhang, Peng Wang, Yanfeng Wu, Huiyong Shen

**Affiliations:** 1 Department of Orthopedics, The Eighth Affiliated Hospital, Sun Yat-sen University, 3025# Shennan Road, 518000 Shenzhen, P.R. China; 2 Center for Biotherapy, The Eighth Affiliated Hospital, Sun Yat-sen University, 3025# Shennan Road, 518000 Shenzhen, P.R. China

**Keywords:** Mesenchymal stem cells, Transcriptomics

## Abstract

Mesenchymal stem cells (MSCs) are a common kind of multipotent cell in vivo, but their heterogeneity limits their further applications. To identify MSC subpopulations and clarify their relationships, we performed cell mapping of bone-marrow-derived MSCs through single-cell RNA (scRNA) sequencing. In our study, three main subpopulations, namely, the stemness subpopulation, functional subpopulation, and proliferative subpopulation, were identified using marker genes and further bioinformatic analyses. Developmental trajectory analysis showed that the stemness subpopulation was the root and then became either the functional subpopulation or the proliferative subpopulation. The functional subpopulation showed stronger immunoregulatory and osteogenic differentiation abilities but lower proliferation and adipogenic differentiation. MSCs at different passages or isolated from different donors exhibited distinct cell mapping profiles, which accounted for their corresponding different functions. This study provides new insight into the biological features and clinical use of MSCs at the single-cell level, which may contribute to expanding their application in the clinic.

## Stem cells: Three subpopulations found in bone marrow

Stem cells in the bone marrow are not all the same: a team led by Huiyong Shen and Yanfeng Wu from Sun Yat-sen University in Shenzhen, China, have identified three main subpopulations of adult stem cells in human marrow, a finding that could lead to more consistent and purer populations of cells for use in in clinical applications. The researchers analyzed gene expression profiles of mesenchymal stem cells (MSCs) using single-cell RNA sequencing methods. The genetic patterns pointed to three distinct cell subpopulations: one that maintained its stem-like capacity for self-renewal; a second “functional” group with strong bone- and blood-forming potential; and a third characterized by active proliferation and cell cycle progression. The authors propose that protocols that isolate only the functional subpopulation of cells could help reduce a source of variation that has limited the therapeutic efficacy of MSC-based therapies.

## Introduction

Mesenchymal stem cells (MSCs), which were first identified in 1976 by Friedenstein^1^, are mainly located in the bone marrow and act as niche cells to support hematopoietic stem cells^2^. Based on subsequent research, MSCs are considered to be multipotent, including exerting immunoregulatory functions and exhibiting a trilineage differentiation capability, which suggests their potential for wide use in tissue regeneration and disease treatment^3^. However, MSCs are a group of mixed cells, and their relatively low purity limits further applications in the clinic. Therefore, identifying the subgroups of MSCs, clarifying their different functions and exploring their relationships are of great significance for basic research and clinical use of MSCs.

Single-cell RNA (scRNA) sequencing has greatly improved during the last decade and is now a well-established and powerful technology in scientific research. Avoiding sample heterogeneity and the equalization effect of bulk sequencing data, scRNA sequencing can easily identify cell types, statuses, functions, and relationships by scanning the RNA expression profile of one cell in a sample^4^. Recently, scRNA sequencing has provided insights into physiological and pathological processes in different kinds of cells and tissues^5–8^. Moreover, scRNA sequencing has been used for subpopulation and functional studies of stem cells^9^. In-depth exploration of MSC heterogeneity still needs to be conducted.

Herein, we aimed to investigate the functional subpopulations of MSCs with scRNA sequencing. We identified three subgroups including the stemness cluster, functional cluster, and proliferative cluster in one MSC sample and investigated their trajectory relationship. In addition, we compared the scRNA-sequencing data of MSCs collected at different passages or from different donors. This study may provide new insight into the biological features and clinical use of MSCs at the single-cell level.

## Materials and methods

### Ethics statement

This study conformed to the Declaration of Helsinki and was approved by the Ethics Committee of the Eighth Affiliated Hospital, Sun Yat-Sen University, Guangzhou, China. The experiments involving mice were approved by the Institutional Animal Care and Use Committee of Sun Yat-Sen University, Guangzhou, China.

### Cell isolation and culture

A total of 15 healthy volunteers were recruited for MSC acquisition in this study. All volunteers were informed of the possible risks of bone marrow puncture and signed informed consent forms. Bone marrow puncture was performed by the same skilled clinician, and MSCs were immediately isolated and purified from the bone marrow samples using density gradient centrifugation methods. Isolated MSCs were resuspended in Dulbecco’s modified Eagle’s medium (DMEM, GIBCO, USA) supplemented with 10% fetal bovine serum (FBS, GIBCO, USA) and then cultured in 25 cm^2^ flasks at 37 °C in 5% CO_2_. The medium was replaced every 3 days thereafter. When cultures reached 90% confluence, the MSCs were digested using 0.25% trypsin containing 0.53 mM EDTA for single-cell experiments or reseeded in new flasks for further expansion.

Another six healthy volunteers were recruited for peripheral blood mononuclear cell (PBMC) acquisition in this study. PBMCs were isolated by density gradient centrifugation with Ficoll-Paque (General Electric Company, USA) and then cultured in RPMI 1640 medium (GIBCO, USA) containing 10% FBS for experiments.

### scRNA sequencing

scRNA sequencing was performed by CapitalBio Technology. Three MSC samples, MSC1 at passage 1, MSC1 at passage 3, and MSC2 at passage 1, were prepared for scRNA-sequencing assays. MSCs were digested into a single-cell suspension, and the cell density and live cell proportion were detected using a Countess II Automated Cell Counter. MSC samples with a density of 1000 cells per μl and a live cell percentage greater than 95% were used for further scRNA sequencing. Briefly, MSC samples were loaded on a 10X Genomics Chromium single-cell controller to generate single-cell gel beads-in-emulsion (GEMs). The captured cells were lysed, and the released RNA was barcoded through reverse transcription in individual GEMs. Then, a single-cell RNA library was prepared using a Chromium Single Cell 3′ Library & Gel Bead Kit V3 according to the manufacturer’s protocols. After quality control, the cDNA libraries were sequenced using an Illumina NovaSeq 6000 sequencer with a sequencing depth of at least 100,000 reads per cell and a paired-end 150 bp (PE150) reading strategy.

### Single-cell data analysis

Sequenced reads were demultiplexed and aligned to the human hg19 reference genome using CellRanger (version 3.0.2) with default parameters. The cell barcodes and unique molecular identifiers (UMIs) associated with the aligned reads were subjected to correction and filtering, and the gene barcode matrix of each MSC sample was constructed. Further analysis, including data normalization, cell cycle correction, dimensionality reduction, and K-means cell clustering, was performed using R (version 3.5.2) with the Seurat package (version 3.0). Gene Ontology (GO) and Kyoto Encyclopedia of Genes and Genomes (KEGG) analyses were performed using DAVID Bioinformatics Resources (version 6.8). A single-cell trajectory analysis was built with Monocle (R package), which introduced pseudotime. Genes were filtered with the following criteria: expressed in more than 10 cells, an average expression value greater than 0.1, and a Qval less than 0.01 in different analyses.

### Flow cytometry and cell sorting

For CD26^+^ and chemokine-like receptor-1^+^ (CMKLR1^+^) MSC sorting, MSCs were digested into single-cell suspensions and separately incubated with an anti-human CMKLR1 PE-conjugated antibody (R&D, USA) or anti-human CD26 FITC-conjugated antibody (BD, USA) for 30 min. After washing with phosphate-buffered saline (PBS) three times, CD26^+^ MSCs or CMKLR^+^ MSCs were isolated with a BD Influx cell sorter for later experiments according to the manufacturer’s instructions. MSCs incubated with PE- or FITC-conjugated IgG antibodies were used as negative controls.

To detect the positive rates of CD26 and CMKLR1 expression, MSCs were then incubated with an anti-human CMKLR1 PE-conjugated antibody (R&D, USA) and anti-human CD26 FITC-conjugated antibody (BD, USA) as described above and detected using a BD Influx cell sorter.

To detect cell markers of MSCs, MSCs were incubated with anti-human CD29 PE-conjugated, anti-human CD44 FITC-conjugated, anti-human CD105 FITC-conjugated, anti-human CD14 FITC-conjugated, anti-human CD34 PE, and anti-human CD45 PE-conjugated antibodies (all from BD Pharmingen, USA) as described above and then detected using a BD Influx cell sorter.

### Cell proliferation assay

MSCs were seeded in 96-well plates at a density of 3000 cells per well. After culturing for 1, 3, 5, 7, 9, or 11 days, the medium was removed, and the cells were incubated in 100 μl fresh serum-free medium containing 10 μl WST-8 (Cell Counting Kit-8, Dojindo, Japan) at 37 °C for 4 h. The absorbance at 450 nm was measured by a Varioskan Flash Spectral Scanning Multimode Reader (Thermo Fisher Scientific Inc, USA). Medium without cells was used as a negative control.

### Quantitative real-time PCR

Total RNA was isolated from MSCs using TRIzol (Invitrogen, USA) and then reverse transcribed into cDNA using a PrimeScript RT reagent kit (Takara, Japan) according to the kit protocol. Quantitative real-time PCR was performed on a LightCycler 480 PCR System (Roche, Switzerland) using SYBR Premix Ex Taq (Takara, Japan). The relative expression levels of each gene were analyzed using the 2^−△△Ct^ method. The forward and reverse primers for each gene are shown in Supplementary Table 1.

### Immunosuppressive function assay

To detect the immunosuppressive capacity of MSCs, specifically the ability to inhibit PBMC proliferation, MSCs were seeded in a 12-well plate at a density of 5 × 10^4^ cells per well. PBMCs were incubated with carboxyfluorescein diacetate *N*-succinimidyl ester (CFSE) at a concentration of 2.5 μM for 10 min at 37 °C and then cocultured in wells with MSCs at a ratio of 10:1. After 5 days of coculture, the nonadherent PBMCs were collected, and then the fluorescence intensity representing the proliferation of PBMCs was detected using a BD Influx cell sorter.

### Osteogenic and adipogenic differentiation

For osteogenic differentiation, MSCs were seeded in 12-well plates at a density of 5 × 10^4^ cells per well in DMEM supplemented with 10% FBS. At 80% confluence, the medium was changed to osteogenic differentiation medium, which consisted of DMEM supplemented with 10% FBS, 100 IU/ml penicillin, 100 IU/ml streptomycin, 0.1 μM dexamethasone, 10 mM β-glycerol phosphate, and 50 μM ascorbic acid (Sigma–Aldrich, USA). The cells were cultured in osteogenic differentiation medium for up to 14 days, and the medium was replaced every 3 days.

For adipogenic differentiation, MSCs were seeded as described, and the medium was changed to adipogenic differentiation medium, which consisted of DMEM supplemented with 10% FBS, 100 IU/ml penicillin, 100 IU/ml streptomycin, 0.5 mM 3-isobutyl-1-methylxanthine, 0.2 mM indomethacin, 10 mg/mL insulin, and 1 mM dexamethasone (Sigma–Aldrich, USA). The medium was replaced every 3 days for 14 days.

### Alizarin Red S (ARS) staining and quantification

On Day 14 of osteogenic induction, MSCs were fixed with 4% paraformaldehyde for 30 min and then stained with 1% ARS (pH 4.3) for 15 min. After washing three times with deionized water, the calcium deposits were observed and imaged with a Nikon Eclipse Ti-S inverted microscope. To quantify calcium deposits, the stained cells were destained with 10% cetylpyridinium chloride monohydrate (CPC, Sigma–Aldrich, USA) in 10 mM sodium phosphate (pH = 7.0) for 1 h. A 200 μl aliquot was transferred to a 96-well plate, and the absorbance was measured at 562 nm by a Varioskan Flash Spectral Scanning Multimode Reader (Thermo Fisher Scientific Inc, USA).

### Alkaline phosphatase (ALP) activity and staining

Intracellular ALP activity was detected by using an ALP activity kit (Nanjing Jiancheng Biotech, China) according to the kit protocol. Briefly, cells were lysed in RIPA lysis buffer (Thermo Fisher, USA) containing protease and phosphatase inhibitors (Roche, Switzerland) on Day 14 of osteogenic induction. The lysate was centrifuged at 12,000 rpm and 4 °C for 30 min, and the supernatant was incubated with reaction buffer at 37 °C for 15 min. Color development was stopped with a stop solution, and the absorbance was measured at 405 nm. The protein concentration of the lysate was determined with a Pierce BCA protein assay kit (Thermo Fisher, USA) according to the kit protocol. ALP activity is expressed as units per gram protein per 15 min (U/g pro/15 min).

An ALP staining assay was performed by using an ALP kit (Sigma–Aldrich, USA). MSCs were fixed with a citrate-acetone-formaldehyde fixative solution for 1 min and then incubated with an alkaline dye solution composed of an FBB-alkaline solution, a sodium nitrite solution and a naphthol AB-BI alkaline solution for 15 min in the dark. The histochemical detection of ALP was observed and imaged with a Nikon Eclipse Ti-S inverted microscope.

### Oil Red O (ORO) staining and quantification

ORO was dissolved in isopropyl alcohol at a concentration of 12 mM and then diluted with ultrapure water at a ratio of 3:2 to obtain a working solution. After adipogenic differentiation induction for 14 days, MSCs were fixed with 4% paraformaldehyde for 20 min and then stained with the ORO working solution for 20 min at room temperature. After three washes with PBS, the stained lipid droplets were observed using a Nikon Eclipse Ti-S inverted microscope. The stained cells were destained thoroughly with 600 ml isopropyl alcohol. A 200 ml aliquot was transferred to a 96-well plate, and the absorbance was measured at 520 nm.

### In vivo bone formation assays

MSCs underwent osteogenic differentiation induction for 14 days, and then a total of 5 × 10^5^ MSCs were loaded onto 40 mg hydroxyapatite/tricalcium phosphate (HA/TCP; Zimmer, USA) and implanted subcutaneously into the dorsal sides of 8-week-old female BALB/c-nu/nu mice (Laboratory Animal Center of Sun Yat-Sen University). Operations were performed under anesthesia administered by intraperitoneal injection of ketamine and xylazine. After 8 weeks, the mice were sacrificed and the implants were harvested.

### In vivo adipose tissue formation assays

For in vivo adipose tissue formation assays, MSCs were cultured under adipogenic differentiation conditions as described above and then mixed with 150 μL Matrigel (BD, USA). The mixture was then injected into the dorsal sides of 8-week-old female BALB/c-nu/nu mice (Laboratory Animal Center of Sun Yat-Sen University). Operations were performed under anesthesia as described above, and the implants were harvested after 8 weeks.

### Hematoxylin and eosin (H&E) and Masson trichrome staining

Tissue samples were fixed with 4% polyoxymethylene for 24 h and then embedded in paraffin for sectioning. HA/TCP implants were decalcified in 20% EDTA for 14 days before paraffin embedding. Sections were stained with hematoxylin for 5 min. After washing with PBS for 10 min, the sections were stained with eosin for 3 min. For Masson trichrome staining, sections were stained using a Masson trichrome staining kit (Sigma–Aldrich, USA) according to the manufacturer’s protocol. All sections were dehydrated and observed using a Nikon Eclipse Ti-S inverted microscope.

### Tissue immunohistochemical assay

Sections were incubated in 10 mM citrate buffer and microwaved at 750 W for 30 min for antigen retrieval. The sections were treated with 3% H_2_O_2_ for 20 min and blocked with 5% normal goat serum for 1 h. Then, the sections were separately incubated with an anti-OCN or anti-Perilipin-1 antibody at 4 °C overnight. Secondary antibody incubation and color development were performed using a SP Rabbit & Mouse HRP DAB Kit (Cwbio) according to the kit protocol. OCN or Perilipin-1 expression was observed using a Nikon Eclipse Ti-S inverted microscope.

### Senescence-associated beta-galactosidase (SA-β-Gal)staining

SA-β-Gal staining was performed using a Senescence-Associated β-gal Assay Kit (Beyotime Biotechnology, China) according to the manufacturer’s instructions. Briefly, MSCs at passage 1 or 3 were seeded in six-well plates for 24 h. The MSCs were washed with PBS and then fixed with a fixation solution at room temperature for 15 min, followed by incubation with a freshly prepared SA-β-gal staining solution in an incubator at 37 °C with low CO_2_ for 18 h. Senescent cells were observed under a Nikon Eclipse Ti-S inverted microscope.

### Statistical analysis

All the results were determined based on at least three independent experiments containing at least triplicate samples. All data are expressed as the means ± standard deviations. Statistical analysis was performed with SPSS (SPSS Inc.). The n values indicate the numbers of individuals in each experiment. *P*-values less than 0.05 were considered statistically significant.

## Results

### scRNA sequencing of MSCs identified distinct subpopulations

**Fig. 1:**
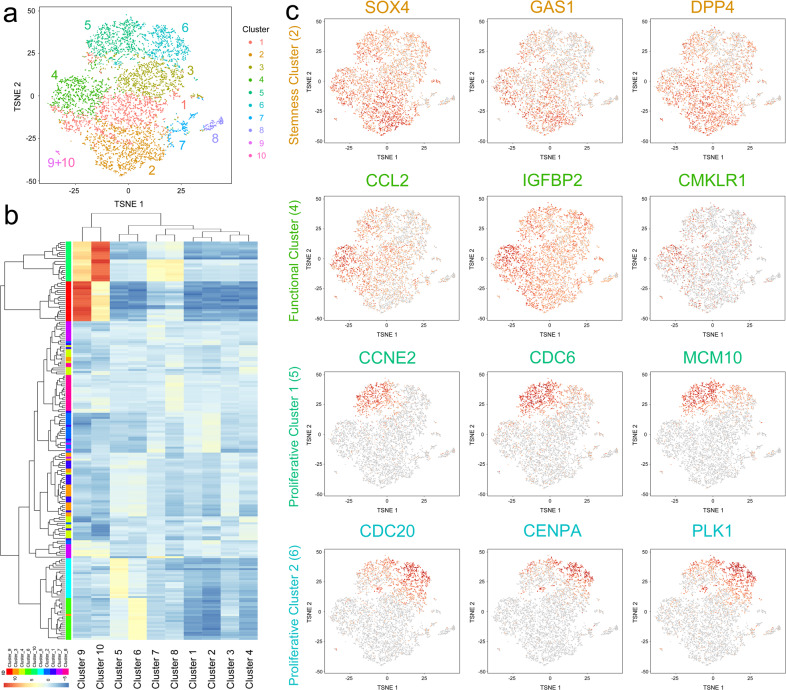
scRNA sequencing of MSCs identified distinct subpopulations. **a** Single-cell sequencing of 6998 bone-marrow-derived MSCs at passage 1 and identification of ten clusters. **b** Hierarchical cluster analysis of the ten identified clusters. **c** Dotplots showing the expression of the marker genes in the four critical clusters on the t-SNE map.

MSCs at passage 1 isolated from the bone marrow of a healthy donor were evaluated using 10X Genomics scRNA sequencing, and cell quality was satisfactory for further analysis (Supplementary Fig. 1). The MSCs were positive for CD29, CD44, and CD105 and negative for CD14, CD34, and CD45, as shown by scRNA-sequencing and flow cytometry data (Supplementary Fig. 2). A total of 6998 cells were captured and then divided into 10 clusters using K-means methods, the cell numbers of which were 1454 (20.78%), 1439 (20.56%), 1107 (15.82%), 931 (13.30%), 900 (12.86%), 734 (10.49%), 243 (3.47%), 154 (2.20%), 30 (0.43%), and 6 (0.09%) (Fig. 1a). Hierarchical cluster analysis showed close relations among these 10 clusters (Fig. 1b). Being identified as monocytes/macrophages from the bone marrow, Clusters 9 and 10, which were characterized by CD14 and CD163 expression, had distinct RNA profiles and relatively independent locations (Fig. 1a, b). In addition, Clusters 1 and 3, which had fewer marker genes, as well as Clusters 7 and 8, which had no marker genes, were considered to be transitional subpopulations. Moreover, Cluster 2, which showed higher levels of stemness markers, including SOX4, GAS1, and DPP4, was predicted to be the stemness subpopulation, and Cluster 4, which exhibited expression of cytokines and osteogenic/adipogenic factors, was identified as the functional subpopulation (Fig. 1c). Clusters 5 and 6, which both exhibited expression of proliferative and cell-cycle-related genes but were divided into the two clusters, were classified as proliferative subpopulations 1 and 2 (Fig. 1c).

### GO and KEGG analyses of MSC subpopulations

**Fig. 2:**
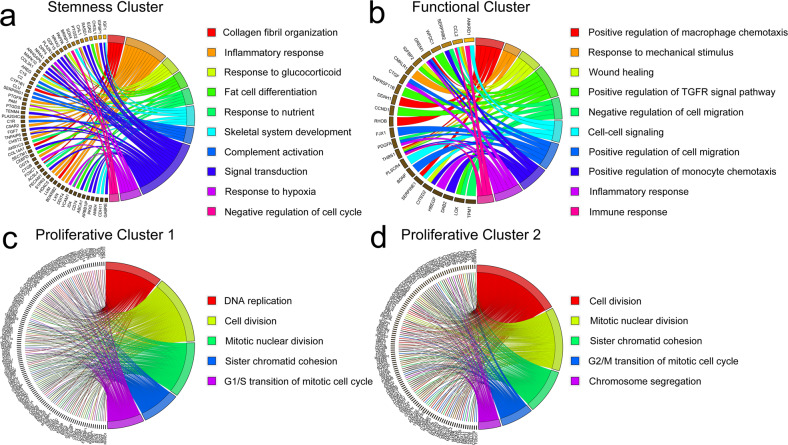
GO and KEGG analyses of MSC subpopulations. **a** The biological process terms for the stemness subpopulation determined by GO analysis. **b** The biological process terms for the functional subpopulation determined by GO analysis. **c** The biological process terms for proliferative subpopulation 1 determined by GO analysis. **d** The biological process terms for proliferative subpopulation 2 determined by GO analysis.

To investigate the possible functions of these identified subpopulations, GO and KEGG analyses were performed (Fig. 2 and Supplementary Fig. 3–6). For the biological process category, the marker genes of the stemness subpopulations were enriched in the inflammatory response, fat cell differentiation, skeletal system development, and negative regulation of the cell cycle, indicating the characteristics of stemness maintenance and pluripotent differentiation in these subpopulations (Fig. 2a). For the functional subpopulation, its marker genes were mainly enriched in several regulatory terms, as well as inflammatory and immune responses (Fig. 2b). Moreover, the G1/S transition of the mitotic cell cycle was enriched in the biological process category of proliferative subpopulation 1, and the G2/M transition of the mitotic cell cycle was enriched in that of proliferative subpopulation 2, which indicated the different stages of these two proliferative clusters in the cell cycle (Fig. 2c, d and Supplementary Table 2 and 3).

### Single-cell trajectory branch analysis of MSC subpopulations

**Fig. 3:**
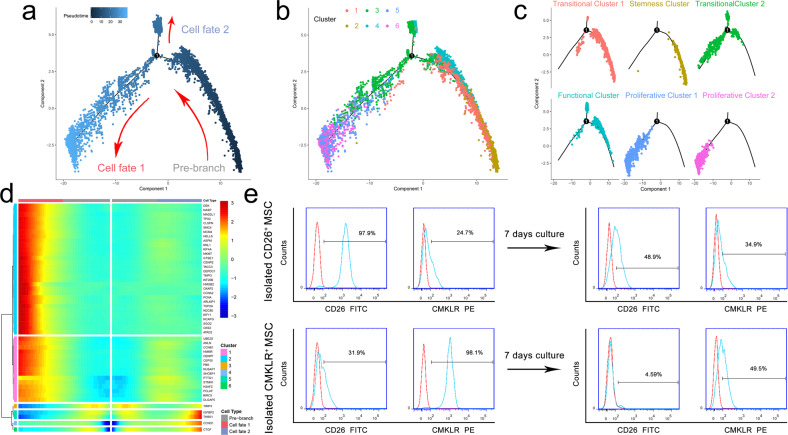
Single-cell trajectory branch analysis of MSC subpopulations. **a**–**c** Monocle pseudotime trajectory branch showing the changing progression of Clusters 1–6. **d** The expression of genes in a branch-dependent manner from the root to fate 1 or 2. **e** The expression levels of CD26 and CMKLR1 in isolated CD26^+^ MSCs and CMKLR1^+^ MSCs before and after 7 days of culture.

Then, we selected Clusters 1–6 to construct a trajectory branch that contained two termini corresponding to two distinct cell fates (Fig. 3a). As shown in Fig. 3b and c, transitional subpopulation 1 (Cluster 1) and the stemness subpopulation (Cluster 2) were located in the root of the trajectory. In addition, the proliferative subpopulations were both located in the cell fate 1 root. The cell fate 2 root was populated by part of transitional subpopulation 2 (Cluster 3) and the functional subpopulation (Cluster 4). Cell-proliferation-related genes, such as MKI67, MCM4, and PCNA, were highly expressed in cell fate 1, and the levels of IGFBP2, CTGF, and TIMP3 were upregulated in cell fate 2 but downregulated in cell fate 1 (Fig. 3d). Then, we confirmed the differentiation relation between the stemness subpopulation and functional subpopulation, separately marked by their feature phenotypes CD26^+^ and CMKLR1^+^. After 7 days of culture, the CD26^+^ rate of isolated CD26^+^ MSCs decreased, and the CMKLR1^+^ rate increased. Moreover, the CD26^+^ rate was low in isolated CMKLR1^+^ MSCs and further decreased after 7 days of culture (Fig. 3e).

### Immunoregulatory potential of the CMKLR1^+^ MSC functional subpopulation

**Fig. 4:**
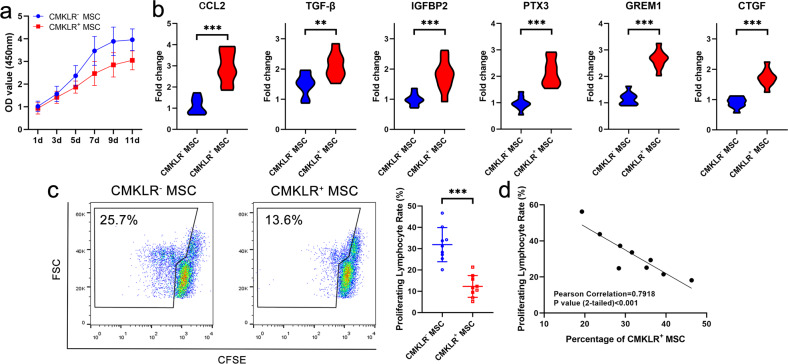
Immunoregulatory potential of the CMKLR1^+^ MSC functional subpopulation. **a** The proliferative ability of CMKLR1^+^ MSCs was weaker than that of CMKLR1^−^ MSCs. **b** PCR results showed that CCL2, TGF-β, IGFBP2, PTX3, GREM1, and CTGF expression levels were higher in CMKLR1^+^ MSCs than in CMKLR1^−^ MSCs. **c** The immunoregulatory ability of CMKLR1^+^ MSCs determined by CFSE-based flow cytometry assays was stronger than that of CMKLR1^−^ MSCs. **d** The immunoregulatory ability of MSCs determined by CFSE-based flow cytometry assays was positively related to the percentage of CMKLR1^+^ MSCs.

MSCs were divided into the CMKLR1^+^ MSC functional subpopulation and the CMKLR1^−^ MSC subpopulation, which included the stemness and proliferative subpopulations, by flow cytometry to further investigate their potential. The results showed that the CMKLR1^+^ MSC functional subpopulation had a lower proliferative rate than the population containing the other MSCs (Fig. 4a). Consistent with the scRNA-sequencing results, immune-related cytokines, including CCL2, TGF-β, IGFBP2, and PTX3, were highly expressed in the CMKLR1^+^ MSC functional subpopulation. In addition, the expression of osteogenesis- and adipogenesis-related factors, such as GREM1 and CTGF, were upregulated in the CMKLR1^+^ MSC functional subpopulation (Fig. 4b). Moreover, the immunoregulatory potential of the CMKLR1^+^ MSC functional subpopulation related to inhibiting PBMC proliferation was stronger than that of the CMKLR1^−^ MSC subpopulation (Fig. 4c). This immunoregulatory potential of the MSC sample was positively correlated with the percentage of CMKLR1^+^ MSCs in the sample (Fig. 4d).

### Differentiation potential of the CMKLR1^+^ MSC functional subpopulation

**Fig. 5:**
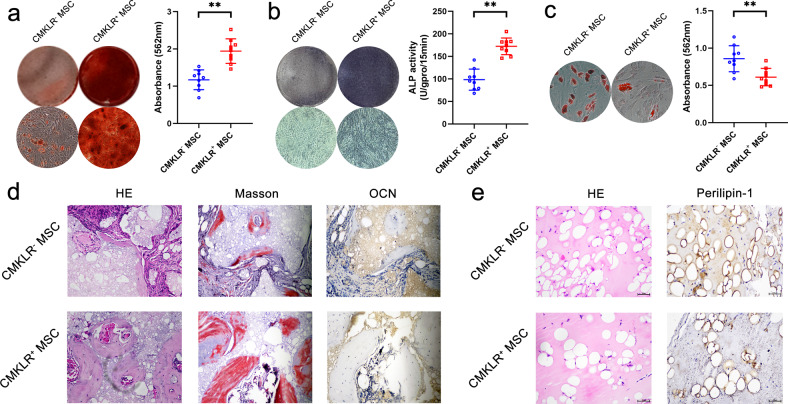
Differentiation potential of the CMKLR1^+^ MSC functional subpopulation. **a** ARS results showed that the osteogenic differentiation ability of CMKLR1^+^ MSCs was stronger than that of CMKLR1^−^ MSCs. **b** ALP assays showed that the osteogenic differentiation ability of CMKLR1^+^ MSCs was stronger than that of CMKLR1^−^ MSCs. **c** ORO staining and quantitative results demonstrated that the adipogenic differentiation ability of CMKLR1^+^ MSCs was weaker than that of CMKLR1^−^ MSCs. **d** H&E and Masson staining showed that more new bone formation was observed in the CMKLR1^+^ MSC group than in the CMKLR1^−^ MSC group in vivo. More OCN^+^ osteoblasts were also observed in the CMKLR1^+^ MSC group than in the CMKLR1^−^ MSC group in vivo. **e** H&E staining and perilipin-1 staining showed that the number of adipocytes in the CMKLR1^+^ MSC group was less than that in the CMKLR1^−^ MSC group in vivo.

Then, we further investigated the osteogenic and adipogenic differentiation potential of the CMKLR1^+^ MSC functional subpopulation. ARS staining results showed that the mineralization level of CMKLR1^+^ MSCs was higher than that of CMKLR1^−^ MSCs, indicating a stronger osteogenic differentiation potential for CMKLR1^+^ MSCs (Fig. 5a). ALP staining and a quantitative assay showed consistent results (Fig. 5b). In contrast, ORO staining showed fewer lipid droplets in CMKLR1^+^ MSCs than in CMKLR1^−^ MSCs, indicating a weaker ability to undergo adipogenic differentiation (Fig. 5c). Then, the osteogenic and adipogenic differentiation abilities of CMKLR1^+^ MSCs were evaluated using in vivo models. Both Masson staining and H&E staining showed that the amount of new bone formation in the CMKLR1^+^ MSC group was much greater than that in the CMKLR1^−^ MSC group, which was also confirmed by the numbers of OCN^+^ osteoblasts in tissue (Fig. 5d). As shown by H&E staining, the in vivo adipocyte formation of the CMKLR1^+^ MSC group was lower than that of the CMKLR1^−^ MSC group. Moreover, there were fewer Perilipin-1^+^ adipocytes in the CMKLR1^+^ MSC group than in the CMKLR1^-^ MSC group (Fig. 5e).

### Comparison of the scRNA-sequencing profiles of MSCs at different passages

**Fig. 6:**
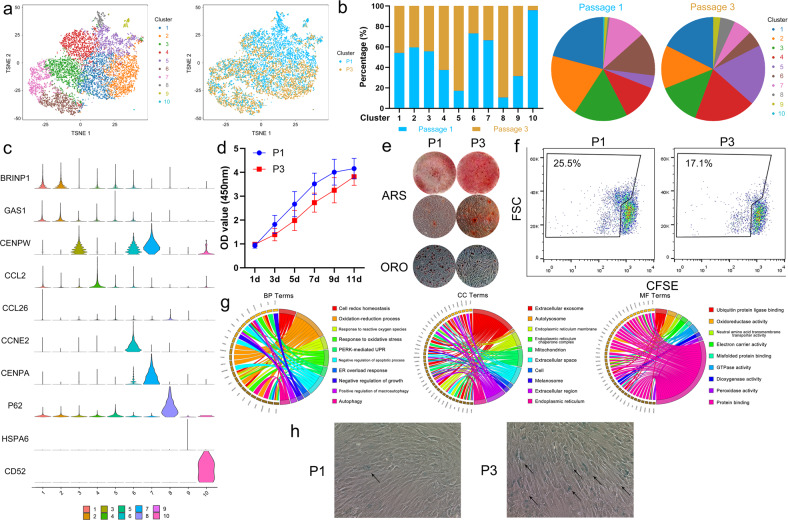
Comparison of the scRNA-sequencing profiles of MSCs at different passages. **a** Merged single-cell sequencing data for bone-marrow-derived MSCs at passages 1 and 3. **b** The percentages of MSCs at passage 1 or 3 in the ten clusters. **c** The violin plot shows the marker gene expression of each cluster for all ten clusters. **d** A CCK-8 assay determined that MSCs at passage 1 had a stronger proliferative ability than those at passage 3. **e** MSCs at passage 3 showed a stronger osteogenic differentiation ability but a weaker adipogenic differentiation ability than MSCs at passage 1. **f** The immunoregulatory ability of MSCs at passage 3 was stronger than that of MSCs at passage 1. **g** The biological process, molecular function, and cellular component terms of Cluster 8 were determined by GO analysis. **h** SA-β-Gal staining showed that the number of senescent MSCs was larger in the passage 3 population than in the passage 1 population.

Using CellRanger AGGR analysis, we compared scRNA-sequencing data between passage 1 and passage 3 of the same MSC sample. As shown in Fig. 6a and b, the new Clusters 5 and 8 were mainly populated by MSCs at passage 3. In addition, the MSCs at passage 3 had higher rates of inclusion in Clusters 4 and 9 than the MSCs at passage 1. Conversely, the MSCs at passage 1 predominated in Clusters 6, 7, and 10 (Fig. 6a and b). By scanning the marker genes of these 10 clusters, Cluster 10, which was defined by CD14 expression, was identified as monocytes/macrophages. Clusters 1, 5, and 9 had fewer marker genes and were considered to be transitional subpopulations. Clusters 3, 6, and 7, which were characterized by cell-cycle-related gene expression, were identified as the proliferative subpopulation. Cluster 2, which included marker genes such as SOX4, EGR2 and IGFBP1, was identified as the stemness subpopulation, and Cluster 4, which showed expression of CCL2, CTGF, and GREM1, was identified as the functional subpopulation. Cluster 8, which showed expression of P62, was newly identified and mainly populated by MSCs at passage 3 (Fig. 6c). The proliferation rate of the MSCs at passage 1 was higher than that of the MSCs at passage 3 (Fig. 6d). Interestingly, the osteogenic differentiation ability of the MSCs at passage 3 was stronger than that of the MSCs at passage 1, but an adipogenic differentiation assay showed the opposite results (Fig. 6e). Moreover, the immunoregulatory potential shown by the CFSE assay was also stronger in the MSCs at passage 3 (Fig. 6f). GO analysis of the marker genes of Cluster 8 showed that this MSC subpopulation was related to cell redox homeostasis, endoplasmic reticulum stress, autophagy, and negative regulation of growth, indicating that this cluster may be a senescent subpopulation (Fig. 6g). Moreover, many more senescent cells were observed in the MSCs at passage 3 than in those at passage 1 (Fig. 6h).

### Comparison of the scRNA-sequencing profiles of MSCs from different donors

**Fig. 7:**
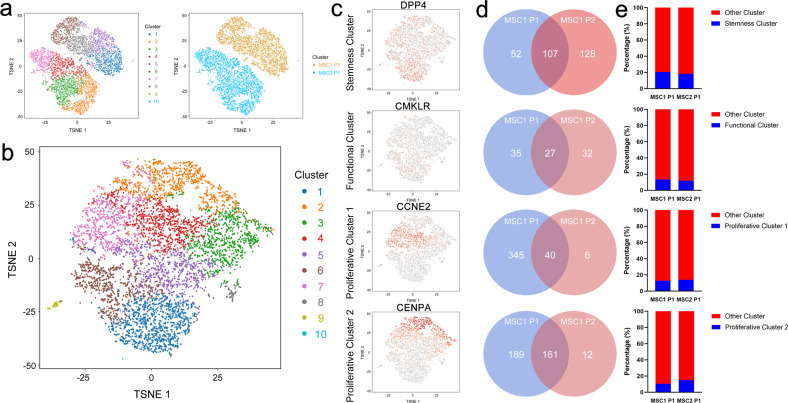
Comparison of the scRNA-sequencing profiles of MSCs from different donors. **a** Merged single-cell sequencing data for bone-marrow-derived MSCs from two different donors (MSC1 P1 & MSC2 P1). **b** Single-cell sequencing of bone-marrow-derived MSC2 at passage 1 and identification of ten clusters. **c** Dotplots showing the expression of the marker genes in the four critical clusters on the t-SNE map. **d** Venn diagram of the marker genes in MSC1 P1 and MSC2 P1. **e** Percentages of subpopulations in MSC1 P1 and MSC2 P1.

When comparing the scRNA-sequencing data of two different MSC samples at the same passage (MSC1 P1 and MSC2 P1), AGGR analysis suggested that the two MSC samples were totally separated into two independent subpopulations without connections (Fig. 7a). Further subpopulation analysis of MSC2 P1 using K-means methods revealed that 10 clusters were identified, matching the number of clusters for MSC1 P1, as shown in Fig. 1 (Fig. 7b). Consistently, a stemness subpopulation marked by DPP4, a functional subpopulation marked by CMKLR, and proliferative subpopulations 1 and 2 marked by CCNE2 and CENPA were identified (Fig. 7c). Although the marker genes of the subpopulation in MSC2 P1 were not totally identical to those in MSC1 P1, most of the genes were consistently expressed in the same subpopulation between MSC1 P1 and MSC2 P1 (Fig. 7d). In addition, the percentage of each subpopulation was almost equal between MSC1 P1 and MSC2 P1 (Fig. 7e).

## Discussion

In this study, using scRNA sequencing, we identified three clusters of MSCs including the CD26^+^ stemness subpopulation, the CMKLR1^+^ functional subpopulation and the proliferative subpopulation. Through trajectory branch analysis, we determined that the stemness subpopulation separately differentiated into the functional subpopulation or the proliferative subpopulation. Further study demonstrated that the CMKLR1^+^ functional subpopulation had stronger abilities related to immunoregulation and osteogenic differentiation but lower potential for adipogenic differentiation and proliferation. By comparing the scRNA-sequencing data of MSCs at different passages, we surprisingly found that the CMKLR1^+^ functional subpopulation contained a larger proportion of MSCs at passage 3 than at passage 1. In addition, a new subpopulation related to senescent cells was identified in the MSCs at passage 3. Moreover, MSCs from different donors showed similar subpopulations but partial differences in expression profiles identified by scRNA sequencing.

MSCs are powerful multipotent stem cells that have been widely used to treat various diseases in the clinic^10–13^. However, the clinical application effect of MSCs is relatively limited and could be further improved. One of the important reasons for this issue is that MSCs are a group of heterogeneous cells^14,15^. Over the past few years, many studies have investigated the characteristics and functions of MSCs with specific markers^16,17^. However, these studies have focused on one of the clusters of MSCs rather than the whole population and the relationships among the clusters. Recently, scRNA sequencing has made it possible to integrally scan the expression profiles in a cell sample at the single-cell level^4^. Although several studies have investigated the features of MSCs using scRNA sequencing^18,19^, the relatively low number of cells detected in these studies has limited the analysis results and final conclusions for MSCs. Therefore, in this study, we performed scRNA sequencing of MSCs to address this issue.

Although MSCs were positive for CD29, CD44, and CD105 and negative for CD14, CD45, and HLA-DR, which meets the International Society for Cellular Therapy (ISCT) criteria for MSC identification^20^, three clusters were identified through scRNA sequencing and subsequent bioinformatic analysis. The first cluster, which was characterized by the expression of stem cell markers such as CD26 (also named DPP4), SOX4 and GAS1, was determined to be the stemness subpopulation, the marker genes of which were enriched in tissue differentiation and the inflammatory response. In addition, the second cluster, which showed expression of many cytokines, including CCL2 and IGFBP2, was identified as the functional subpopulation, and its marker genes were related to several regulatory processes. Moreover, the third cluster, with relatively high expression of cell-cycle-related genes, was defined as the proliferative subpopulation, which was divided into the G1/S and G2/M subgroups. Specifically, obvious front and back relationships among these three subpopulations were shown by trajectory branch analysis. In addition, isolated CD26^+^ MSCs (the stemness subpopulation) could develop into CMKLR1^+^ MSCs (the functional subpopulation), but the opposite was not observed. Therefore, we concluded that the stemness subpopulation was the base population of MSCs and gradually became the functional subpopulation or proliferative subpopulation.

Although the CD26^+^ stemness subpopulation was found to be fundamental, the functional subpopulation expressing higher levels of various kinds of cytokines attracted our attention. Among the marker genes, CMKLR1 was chosen as the sorting marker for the functional subpopulation. CMKLR1, also known as ChemR23, is a 7-transmembrane G-protein receptor that is widely expressed in various kinds of cells and participates in many physiological processes^21,22^. In our study, from our scRNA-sequencing data and subsequent PCR results, we determined that the CMKLR1^+^ functional subpopulation had high expression of many cytokines, including CCL2, TGF-β, IGFBP2, PTX3, GREM1, and CTGF, which are related to the immunoregulatory and multipotent differentiation abilities of MSCs^23–28^. Moreover, the expression of these cytokines in the CMKLR1^+^ functional subpopulation was much higher than that in the stemness subpopulation according to the scRNA-sequencing results, indicating the more mature functions of the functional subpopulation compared to those of the stemness subpopulation. In addition, functional experiments showed that the CMKLR1^+^ functional subpopulation had stronger immunoregulatory and osteogenic differentiation abilities but lower proliferation and adipogenic differentiation. These results were consistent with a previous study showing that CMKLR1 promoted osteogenesis but inhibited adipogenesis in MSCs^29^. In addition, CMKLR1 knockout mice have a reduced bone mass because of a decrease in osteogenic MSC differentiation^29^. Therefore, we suggest that this CMKLR1^+^ subpopulation may be the main functional subpopulation of MSCs.

Previous studies have demonstrated that MSC functions gradually change with ongoing passaging. At higher passages, MSCs become senescent and show weakening of various abilities, including decreased differentiation and migration^30,31^. However, whether MSCs at passage 1 show the strongest abilities seems to be controversial. Considering that three subpopulations of MSCs exist and that the stemness subpopulation irreversibly becomes the functional subpopulation or proliferative subpopulation, the cell mappings and functions of MSCs at different passages could reflect dynamic changes. To investigate this issue, we compared the scRNA-sequencing data of MSCs at passages 1 and 3. A new subpopulation, the marker genes of which were enriched in oxidative stress, endoplasmic reticulum stress and autophagy, was identified in the MSCs at passage 3. These biological processes were reported to be related to MSC senescence^32–35^, confirming that the proportion of senescent MSCs increased with passaging. Interestingly, the proportion of the functional subpopulation was higher in the MSCs at passage 3 than in the MSCs at passage 1 (62.5% versus 37.4%, respectively). In addition, functional experiments showed that the MSCs at passage 3 exhibited slightly stronger osteogenic differentiation and immunoregulatory activity. Therefore, we suggest that MSC functions mainly depend on the proportion of the functional subpopulation at the timepoint evaluated rather than the number of passages and that MSCs at passage 1 may not be the strongest population because the stemness subpopulation can partially differentiate into the functional subpopulation before becoming exhausted. Moreover, directly isolating the CMKLR1^+^ functional subpopulation or promoting differentiation of the stemness subpopulation into the functional subpopulation may have relatively good application prospects for clinical use.

The cell maps of MSCs from different donors are still unclear. We further compared the scRNA-sequencing data of MSCs from different donors. To our surprise, the two cell samples were separated without connections or overlap in the AGGR analysis. However, further analysis showed that both MSC samples contained a stemness subpopulation, functional subpopulation, and proliferative subpopulation. In addition, most of the marker genes in these subpopulations were the same between the two MSC samples. In addition, the percentages of these three subpopulations were also similar between MSC1 and MSC2 at the same passage. These results suggest that MSCs from different donors have the same composition. Notably, MSCs from different donors show different levels of function and reactions to stimulation^36,37^. Therefore, depending on the differentially expressed marker genes of each MSC sample, individual differences truly exist among MSCs, which could lead to various clinical application effects.

In conclusion, our study determined the scRNA-sequencing profiles of MSCs at different passages or from different donors and revealed three subpopulations including the stemness subpopulation, functional subpopulation and proliferative subpopulation, as well as their relationships. Limitations still existed in our study. What are the divergent genes that affect the changes between the stemness subpopulation and the other two subpopulations? Which factors can increase the proportion of the functional subpopulation? What is the application effect of the functional subpopulation in the clinic? These issues need to be addressed in future studies.

## Supplementary information

Supplemental information file

## Data Availability

The scRNA-sequencing data generated in this study have been deposited in the NCBI database under accession code SUB10399764. Other data in this study are available from the corresponding author upon request.
