# Supplementary material for: Single-cell RNA sequencing analysis of human bone-marrow-derived mesenchymal stem cells and functional subpopulation identification
Source: Exp Mol Med. 2022 Apr 1;54(4):483–92. doi: 10.1038/s12276-022-00749-5 (PMC9076886; doi:10.1038/s12276-022-00749-5)
Supplement: Supplementary file 1 — Supplemental information file [file 12276_2022_749_MOESM1_ESM.pdf]

Supplementary Fig. 1 Cell quality for single-cell sequencing

The UMI and gene counts of the detected cells shown on the t-SNE map.

Supplementary Fig. 2 Cell markers of MSCs in scRNA sequencing and flow cytometry

MSCs were positive for CD29, CD44, and CD105 and negative for CD14, CD34, and CD45.

Supplementary Fig. 3 GO and KEGG analyses of the stemness subpopulation

The biological process, molecular function and cellular component terms identified by GO analysis and the top ten enriched pathways identified by KEGG analysis of the stemness subpopulation.

Supplementary Fig. 4 GO and KEGG analyses of the functional subpopulation

The biological process, molecular function and cellular component terms identified by GO analysis and the top ten enriched pathways identified by KEGG analysis of the functional subpopulation.

Supplementary Fig. 5 GO and KEGG analyses of proliferative subpopulation 1

The biological process, molecular function and cellular component terms identified by GO analysis and the top ten enriched pathways identified by KEGG analysis of proliferative subpopulation 1.

Supplementary Fig. 6 GO and KEGG analyses of proliferative subpopulation 2

The biological process, molecular function and cellular component terms identified by GO analysis and the top ten enriched pathways identified by KEGG analysis of proliferative subpopulation 2.

Supplementary Table 1 Primers for qRT-PCR

Gene	Forward primer (5`-3`)	Reverse primer (5`-3`)
GAPDH	GGAGCGAGATCCCTCCAAAAT	GGCTGTTGTCATACTTCTCATGG
CCL2	CAGCCAGATGCAATCAATGCC	TGGAATCCTGAACCCACTTCT
TGF-β	GTAGCTCTGATGAGTGCAATGAC	CAGATATGGCAACTCCCAGTG
IGFBP2	GACAATGGCGATGACCACTCA	CAGCTCCTTCATACCCGACTT
PTX3	TTATTCCCAATGCGTTCCAAGA	GCACTAAAAGACTCAAGCCTCAT
GREM1	CGGAGCGCAAATACCTGAAG	GGTTGATGATGGTGCGACTGT
CTGF	CAGCATGGACGTTCGTCTG	AACCACGGTTTGGTCCTTGG

Supplementary table 2

Gene symbol	Log2 Fold Change
FEN1	2.935536
MRE11	1.418175
MCM7	2.373598
MCM8	2.26455
MCM10	4.138915
BRCA1	2.86558
CHAF1A	3.112554
EXO1	4.015916
CHEK1	1.467726
TOPBP1	1.764003
TIPIN	2.139575
SUPT16H	1.039385
RFC3	2.776971
RFC4	2.765397
LIG1	2.027143
RFC1	1.275678
RMI1	2.385752
RFC2	2.011753
DBF4	1.4762
MCM3	2.838499
TIMELESS	1.86632
MCM4	2.666432
MCM5	2.560618
MCM6	2.3425
DTL	3.956429
MCM2	2.955375
RHNO1	0.929518
PCNA	2.220505
POLD3	2.493612
BRIP1	3.413335
ORC6	2.6485
RBBP8	1.852159
CLSPN	3.150775
BARD1	2.44016
GINS2	2.565464
CDT1	3.14733
DUT	1.029468
RRM1	1.594176
RRM2	1.930456
RPA1	1.297289
RPA2	1.602528
SSRP1	0.983949
CDC6	3.905793
FAM111A	1.682143
POLA1	2.226103
POLA2	2.198951
NASP	1.828586
RPA3	0.932672
POLE3	1.1466
CDK1	1.667457
RAD1	1.063463
ERCC6L	2.239518
USP37	1.74614
ZWILCH	1.172221
CDCA7L	2.139075
CCNF	1.047201

NCAPG2	1.460765
BUB1B	1.184632
KIF11	1.435932
SMC3	1.301353
SMC4	1.204918
SMC2	0.987434
CCND3	1.010907
MIS18BP1	1.129577
NUF2	1.122924
KNTC1	1.971914
FBXO5	2.841987
SPDL1	1.391126
HELLS	2.872735
HAUS6	1.524402
VRK1	1.900651
KNL1	1.076291
SMC1A	1.282667
MASTL	2.668911
CCSAP	1.007808
HAUS1	1.114009
SGO1	1.561014
CCNA2	1.30648
SGO2	0.953301
CCNE2	4.251422
KIFC1	1.926731
BIRC5	1.052779
KIF2C	0.960964
KIF20B	1.073865
ITGB3BP	1.374313
CDCA2	1.417041
CDCA4	2.283238
CDCA5	2.597367
NCAPG	1.969285
CDCA8	1.084822
SKA3	2.062131
NCAPH	1.796582
SKA1	1.82844
DSN1	2.303132
MCMBP	1.450455
SAC3D1	1.304699
CENPX	1.02232
RCC2	0.958686
NDC80	1.234302
ZWINT	1.728831
NEDD1	1.316264
WEE1	1.500421
MIS18A	1.652199
CENPJ	1.883941
TACC3	1.328703
NCAPD3	1.65624
SPC25	1.302168
MAD2L1	1.55497
YEATS4	1.836655
KIF15	1.884215
NUP62	1.446928
PBK	1.129277
INCENP	1.031166
NOLC1	1.003147

AURKB	1.808422
ANLN	0.960726
CENPN	1.258183
NUP107	1.169232
CENPU	2.7449
DDX11	2.54839
CENPH	1.42806
CENPK	1.868297
CENPL	1.359069
CENPM	1.640908
PRIM2	1.878187
PRIM1	3.155858
TYMS	1.635421
PPAT	1.271959
EIF4EBP1	0.989518
RANBP1	0.975873
CDKN2C	1.09295
DHFR	1.591592

Supplementary table 3

Gene symb	Log2 Fold Change
ERCC6L	2.620536
ZWILCH	1.569499
CCNF	3.455371
NCAPG2	2.044759
KIF14	3.768227
BUB1B	3.368858
KIF11	3.143151
SMC4	2.496832
CKS1B	2.261582
SMC2	1.472151
CDC20	3.584997
TUBA1C	1.676992
PTTG1	1.995958
MIS18BP1	2.118241
CNTRL	2.253416
NUF2	3.509828
KNTC1	1.742633
NEK2	3.919552
FBXO5	1.563483
KMT5A	1.215264
SPDL1	2.269053
HELLS	1.043674
LIG1	1.539678
TUBB	1.184184
HAUS6	1.074748
VRK1	1.563427
KNL1	3.199171
SMC1A	1.219385
MASTL	1.172901
CCSAP	1.682839
KNSTRN	2.391554
CKAP5	1.890578
CDC25B	2.257686
SGO1	3.167241
CCNA2	3.366578
SGO2	3.238056
KIFC1	2.90998
CKS2	2.928416
TIMELESS	1.491325
BIRC5	2.416937
MCM5	1.144287
KIF2C	3.622585
KIF20B	3.075404
ITGB3BP	1.444958
CDCA2	3.256567
CDCA3	3.11203
CDCA4	1.243772
CDCA5	2.049053
NCAPG	2.73524
CDCA8	3.795958
SKA3	2.311082
NCAPH	2.51443
SKA1	2.816421
AURKA	3.826738
CCNB2	3.015049
CCNB1	3.321124

RAD21	1.697236
BUB3	1.808302
FAM83D	4.224186
BUB1	3.15687
SAC3D1	1.341114
CENPW	1.798517
SPAG5	3.036071
UBE2C	3.753382
NDE1	1.81369
NDC80	3.304675
ZWINT	2.183089
MAD2L2	1.185439
CENPE	3.63912
TPX2	3.204753
NEDD1	1.237917
CENPF	3.427143
WEE1	1.90561
MIS18A	1.913845
UBE2S	2.288222
REEP4	2.065033
CENPJ	1.695594
CDK1	3.185669
TACC3	2.99506
NCAPD2	2.352147
NCAPD3	1.438254
SPC25	2.979352
MAD2L1	2.386446
KIF15	2.89503
PBK	2.854683
CEP55	3.132055
KIF22	1.613923
CIT	2.519706
ASPM	3.82323
INCENP	3.042269
AURKB	3.053953
PLK1	3.961599
ANLN	2.873169
CENPN	1.555091
MRE11	1.091346
NUP107	1.320773
CENPA	4.174137
CENPU	1.51098
DDX11	1.12577
RANGAP1	1.515178
KIF18A	3.367401
CENPH	1.674664
CENPK	2.063573
CENPL	1.930915
CENPM	1.799102
CCP110	1.037986
HMMR	3.33203
FOXM1	2.155673
CEP192	1.225658
PCNT	1.448829
CEP78	1.352013
CEP135	1.132995
ODF2	1.391645
CEP152	1.53284

TUBB4B	1.827119
TUBG1	1.029274
MELK	2.385446
TOP2A	3.586443
HJURP	3.483196
BRCA1	1.112929
ESCO2	1.808569

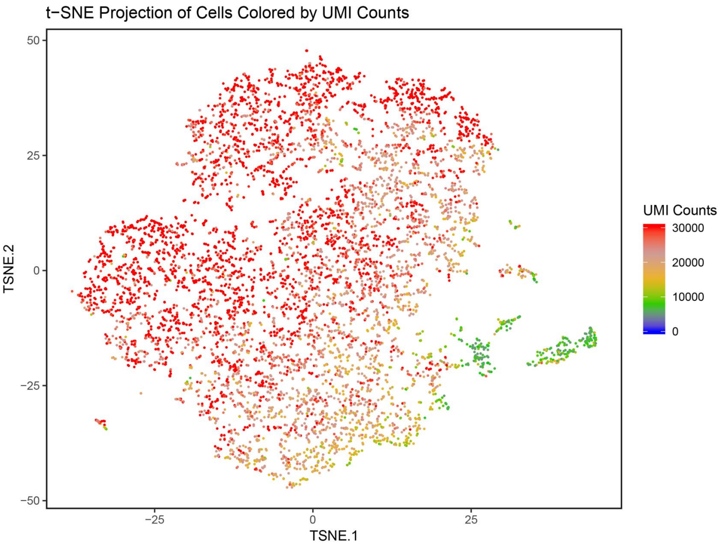
t−SNE Projection of Cells Colored by UMI Counts
50
25
0
−25
−50
TSNE.2
−25
0
25
TSNE.1
UMI Counts
30000
20000
10000
0

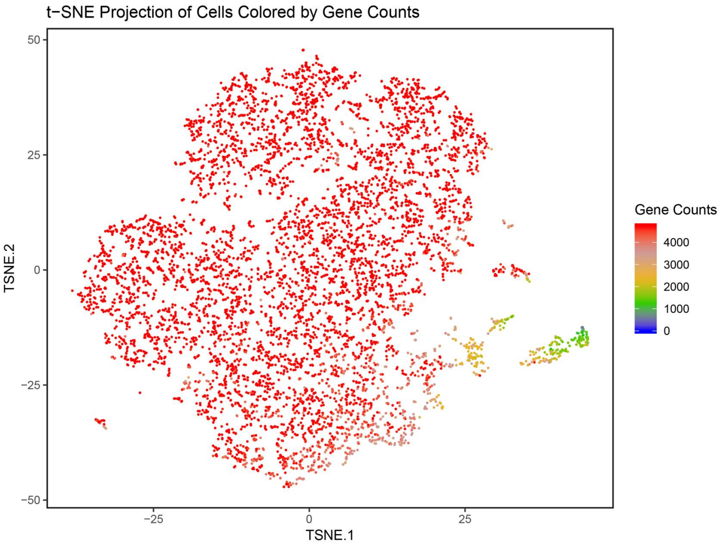
t−SNE Projection of Cells Colored by Gene Counts
50
25
0
−25
−50
TSNE.2
−25
0
25
TSNE.1
Gene Counts
4000
3000
2000
1000
0

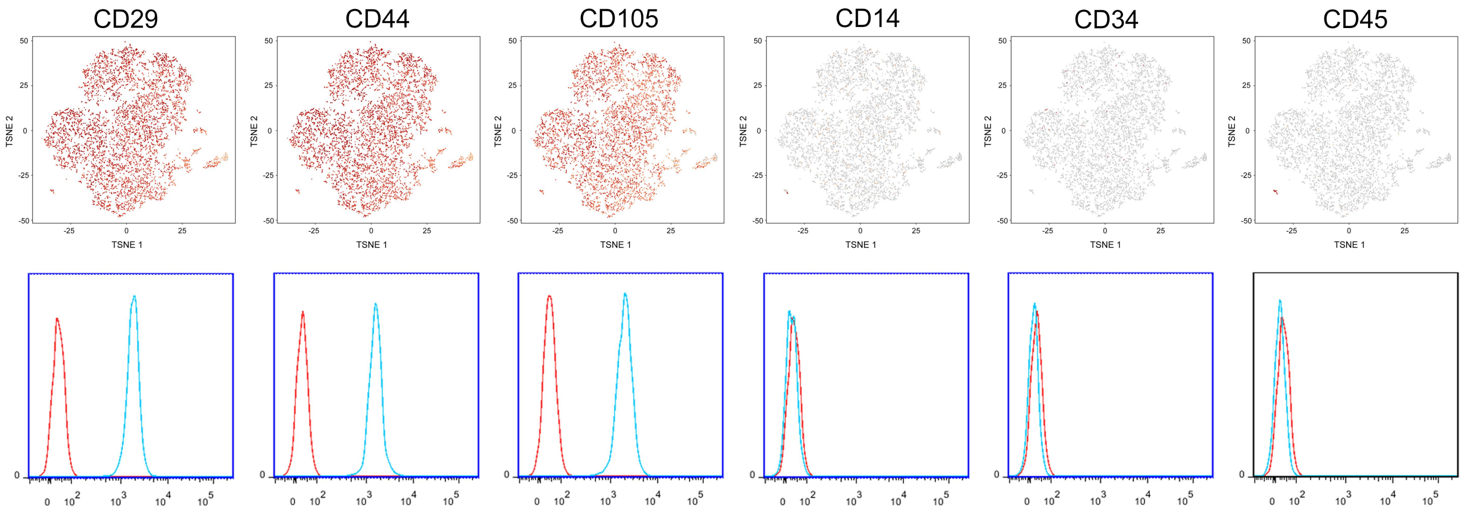

CD29
CD44
CD105
CD14
CD34
CD45
TSNE 1
TSNE 2

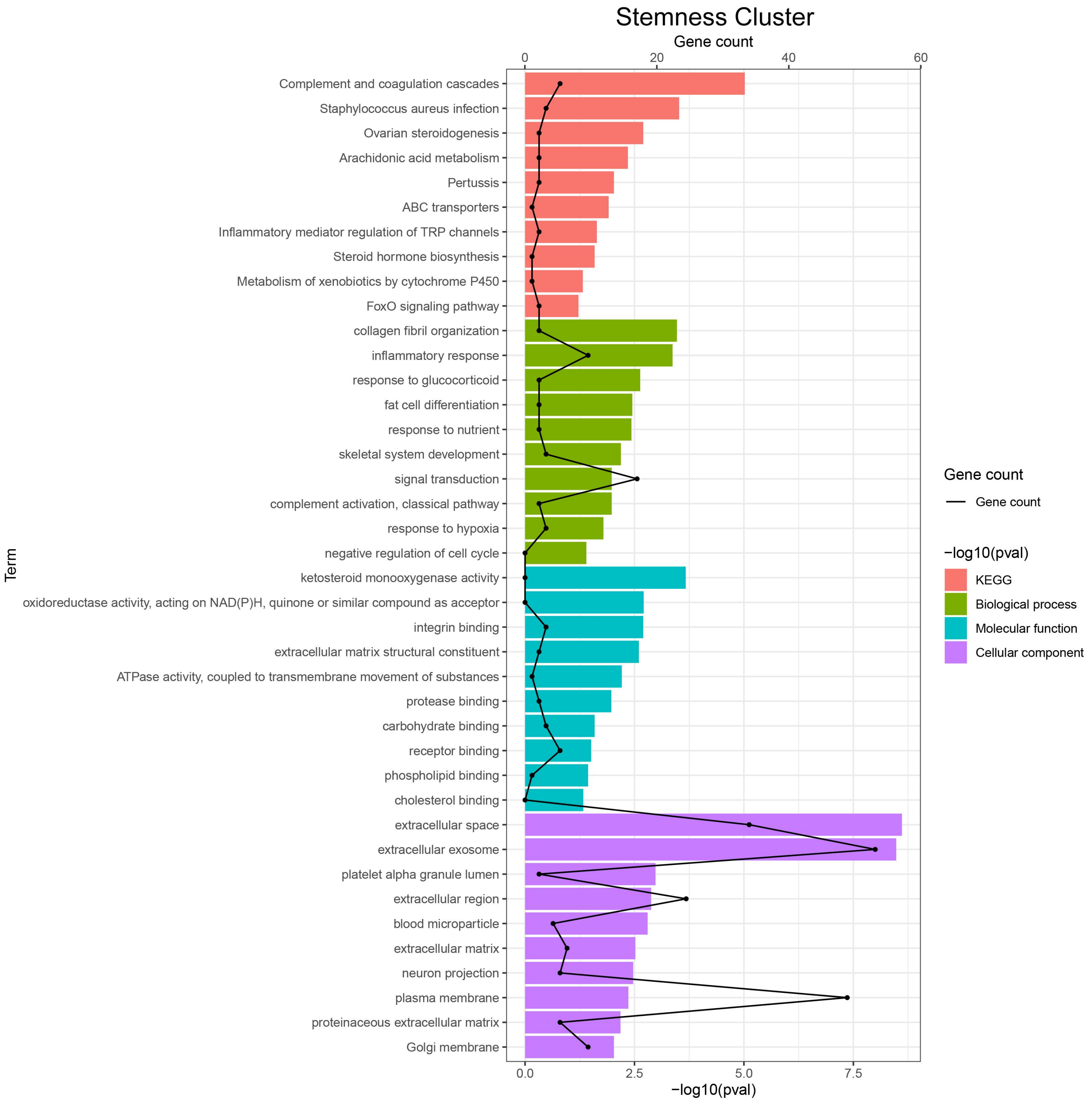
Stemness Cluster
Gene count
0
20
40
60
Complement and coagulation cascades
Staphylococcus aureus infection
Ovarian steroidogenesis
Arachidonic acid metabolism
Pertussis
ABC transporters
Inflammatory mediator regulation of TRP channels
Steroid hormone biosynthesis
Metabolism of xenobiotics by cytochrome P450
FoxO signaling pathway
collagen fibril organization
inflammatory response
response to glucocorticoid
fat cell differentiation
response to nutrient
skeletal system development
signal transduction
complement activation, classical pathway
response to hypoxia
negative regulation of cell cycle
ketosteroid monooxygenase activity
oxidoreductase activity, acting on NAD(P)H, quinone or similar compound as acceptor
integrin binding
extracellular matrix structural constituent
ATPase activity, coupled to transmembrane movement of substances
protease binding
carbohydrate binding
receptor binding
phospholipid binding
cholesterol binding
extracellular space
extracellular exosome
platelet alpha granule lumen
extracellular region
blood microparticle
extracellular matrix
neuron projection
plasma membrane
proteinaceous extracellular matrix
Golgi membrane
Term
0.0
2.5
5.0
7.5
−log10(pval)
Gene count
Gene count
−log10(pval)
KEGG
Biological process
Molecular function
Cellular component

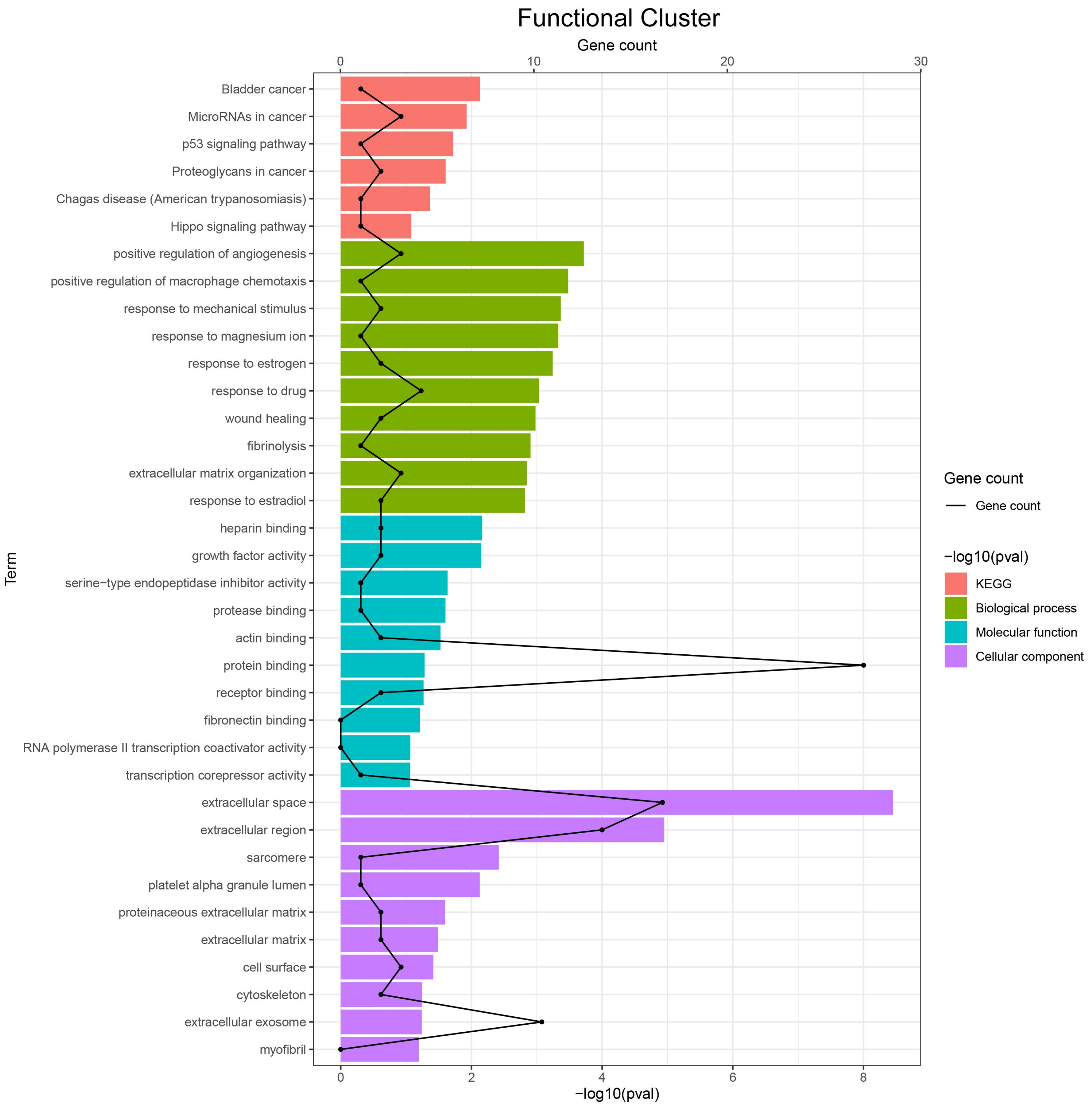
Functional Cluster
Gene count
0
10
20
30
Bladder cancer
MicroRNAs in cancer
p53 signaling pathway
Proteoglycans in cancer
Chagas disease (American trypanosomiasis)
Hippo signaling pathway
positive regulation of angiogenesis
positive regulation of macrophage chemotaxis
response to mechanical stimulus
response to magnesium ion
response to estrogen
response to drug
wound healing
fibrinolysis
extracellular matrix organization
response to estradiol
heparin binding
growth factor activity
serine−type endopeptidase inhibitor activity
protease binding
actin binding
protein binding
receptor binding
fibronectin binding
RNA polymerase II transcription coactivator activity
transcription corepressor activity
extracellular space
extracellular region
sarcomere
platelet alpha granule lumen
proteinaceous extracellular matrix
extracellular matrix
cell surface
cytoskeleton
extracellular exosome
myofibril
Term
0
2
4
6
8
−log10(pval)
Gene count
Gene count
−log10(pval)
KEGG
Biological process
Molecular function
Cellular component

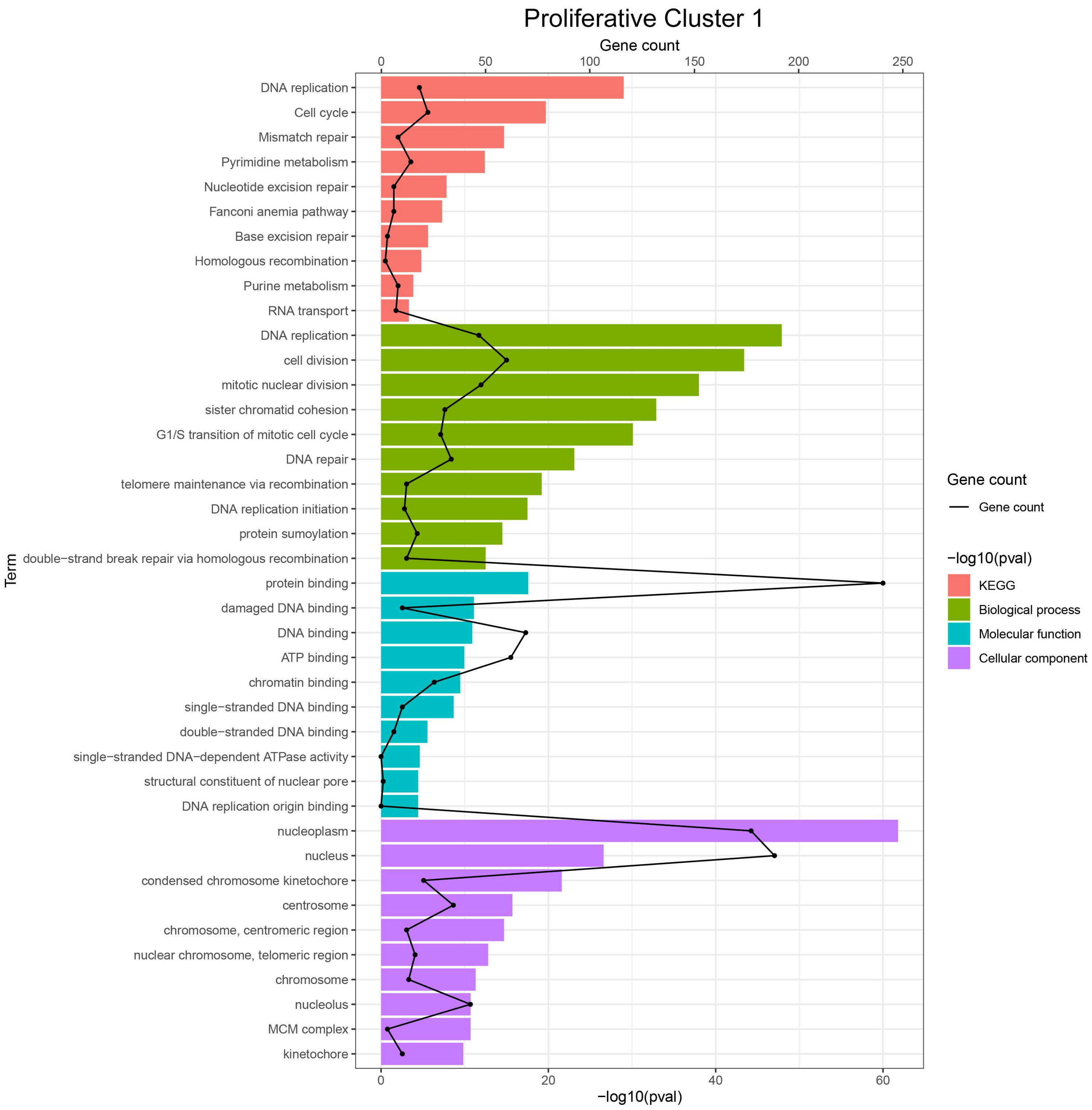

Proliferative Cluster 1
Gene count
0
50
100
150
200
250
Term
DNA replication
Cell cycle
Mismatch repair
Pyrimidine metabolism
Nucleotide excision repair
Fanconi anemia pathway
Base excision repair
Homologous recombination
Purine metabolism
RNA transport
DNA replication
cell division
mitotic nuclear division
sister chromatid cohesion
G1/S transition of mitotic cell cycle
DNA repair
telomere maintenance via recombination
DNA replication initiation
protein sumoylation
double−strand break repair via homologous recombination
protein binding
damaged DNA binding
DNA binding
ATP binding
chromatin binding
single−stranded DNA binding
double−stranded DNA binding
single−stranded DNA−dependent ATPase activity
structural constituent of nuclear pore
DNA replication origin binding
nucleoplasm
nucleus
condensed chromosome kinetochore
centrosome
chromosome, centromeric region
nuclear chromosome, telomeric region
chromosome
nucleolus
MCM complex
kinetochore
0
20
40
60
−log10(pval)
Gene count
Gene count
−log10(pval)
KEGG
Biological process
Molecular function
Cellular component

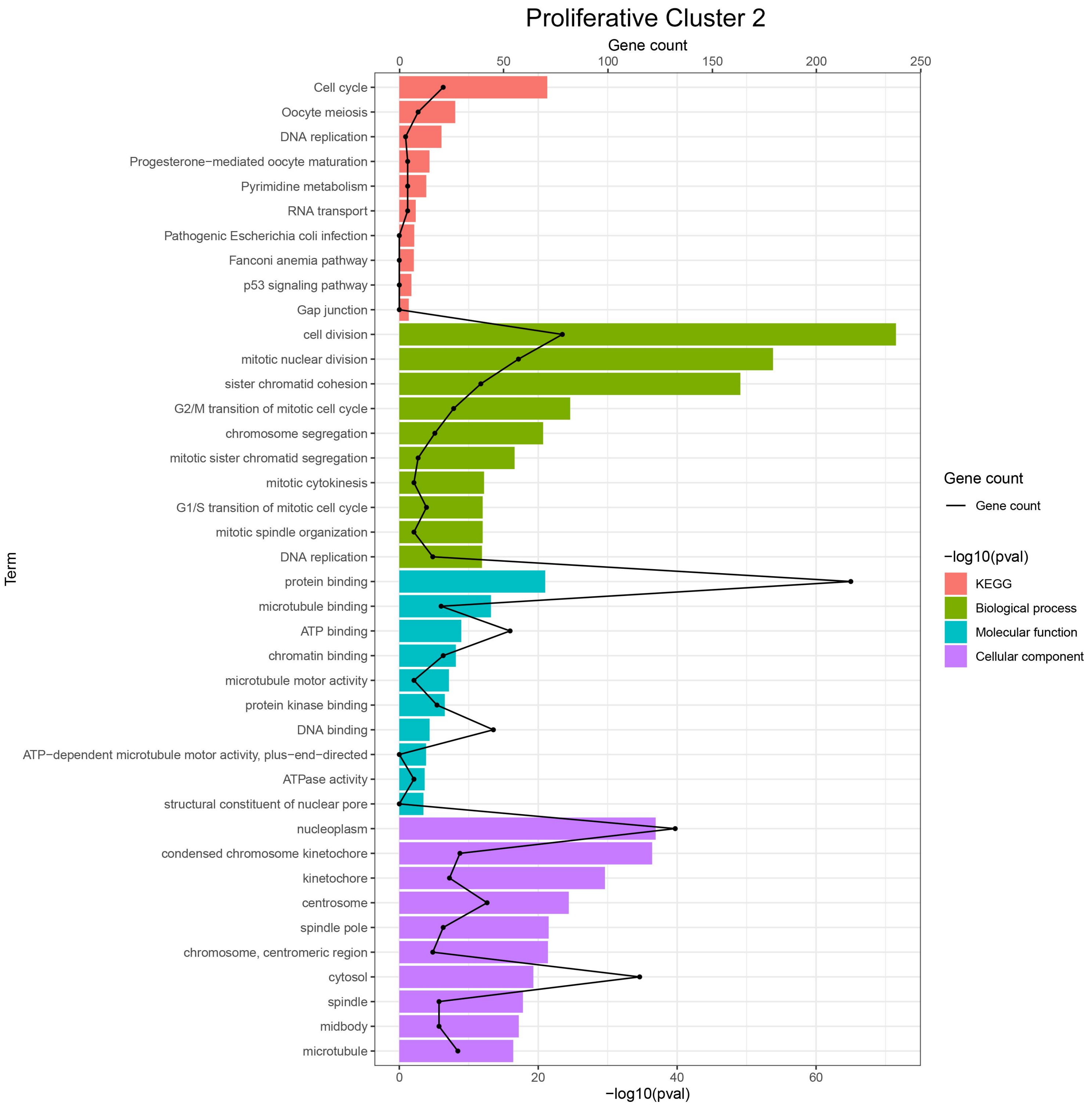

Proliferative Cluster 2
Gene count
0
50
100
150
200
250
Cell cycle
Oocyte meiosis
DNA replication
Progesterone−mediated oocyte maturation
Pyrimidine metabolism
RNA transport
Pathogenic Escherichia coli infection
Fanconi anemia pathway
p53 signaling pathway
Gap junction
cell division
mitotic nuclear division
sister chromatid cohesion
G2/M transition of mitotic cell cycle
chromosome segregation
mitotic sister chromatid segregation
mitotic cytokinesis
G1/S transition of mitotic cell cycle
mitotic spindle organization
DNA replication
protein binding
microtubule binding
ATP binding
chromatin binding
microtubule motor activity
protein kinase binding
DNA binding
ATP−dependent microtubule motor activity, plus−end−directed
ATPase activity
structural constituent of nuclear pore
nucleoplasm
condensed chromosome kinetochore
kinetochore
centrosome
spindle pole
chromosome, centromeric region
cytosol
spindle
midbody
microtubule
Term
0
20
40
60
−log10(pval)
Gene count
Gene count
−log10(pval)
KEGG
Biological process
Molecular function
Cellular component
